# Efficacy and Safety of Drug-Eluting Bead Bronchial Arterial Chemoembolization Plus Anlotinib in Patients With Advanced Non-small-Cell Lung Cancer

**DOI:** 10.3389/fcell.2021.768943

**Published:** 2021-10-29

**Authors:** Juanfang Liu, Wenguang Zhang, Jianzhuang Ren, Zhen Li, Huibin Lu, Zhanguo Sun, Xinwei Han

**Affiliations:** Department of Interventional Radiology, The First Affiliated Hospital of Zhengzhou University, Zhengzhou, China

**Keywords:** anlotinib, non-small cell lung cancer, drug-eluting bead bronchial arterial chemoembolization, combination therapy, overall survival

## Abstract

**Aim:** The aim of this study is to determine the efficacy and safety of the combination therapy of drug-eluting bead bronchial arterial chemoembolization plus anlotinib oral administration in the treatment of non-small-cell lung cancer (NSCLC).

**Methods:** Consecutive data from 51 patients with advanced NSCLC were retrospectively collected from February 2018 to August 2019. All patients underwent drug-eluting bead bronchial arterial chemoembolization (DEB-BACE) followed by anlotinib treatment. Overall survival (OS) and progression-free survival (PFS) were calculated and analyzed using the Kaplan–Meier method and log-rank test, and factors associated with OS and PFS were assessed by a Cox proportional hazards test. Treatment response at 30 days was assessed by enhanced computed tomography (CT), and then the objective response rate (ORR) and disease control rate (DCR) were calculated. Treatment-related adverse events (TRAEs) were also evaluated.

**Results:** The median OS was 18.4 months (95% CI, 16.6–20.2 months), and the median PFS was 8.4 months (95% CI, 6.2–10.6 months). The ORR and DCR for the whole cohort were 21.6 and 100%, respectively, at 30 days after the first cycle of treatment. Most of the treatment-related adverse reactions were mild and moderate and included anorexia, hypertension, fatigue, and hand-foot syndrome. Only eight (15.7%) patients developed grade 3 TRAEs. No deaths or other serious adverse reactions occurred. Both TNM stage and brain metastasis were independent risk factors for OS and PFS.

**Conclusion:** DEB-BACE concomitant with anlotinib has promising efficacy and tolerable toxicity in patients with advanced NSCLC.

## Introduction

Lung cancer is the leading cause of cancer death worldwide, accounting for approximately 18.4% of all cancer deaths (Bray et al., 2018). Non-small-cell lung cancer (NSCLC) is the main histological type of lung cancer, accounting for approximately 83% of lung cancer cases. Due to the absence of specific symptoms at an early stage, most patients are diagnosed at locally advanced or advanced stages (Miller et al., 2016). Chemotherapy with or without radiation remains the main treatment for patients with advanced NSCLC (stage III/IV). Recently, many studies have demonstrated that bronchial artery chemoembolization (BACE) is more effective and less toxic than conventional chemotherapy in treating advanced NSCLC patients (Zhu et al., 2017; Liu et al., 2021). BACE is a combination technique of transcatheter arterial chemical infusion (TAI) followed by embolization, which is performed by injecting chemotherapy drugs and then putting the embolization agents into the tumor-feeding arteries, promoting the clinical efficacy and reducing the systemic toxicity in patients with advanced NSCLC. Given the advantage of slow and sustained release of drugs, drug-eluting bead bronchial artery chemoembolization (DEB-BACE) has been developed and widely used in combination therapy for advanced NSCLC (Bie et al., 2019; Zeng et al., 2020).

Currently, targeted therapy drugs have an obvious advantage in prolonging survival time and have been recommended as a promising therapeutic option for advanced NSCLC patients (Miller et al., 2016; Han et al., 2018). Anlotinib is an oral multitargeted tyrosine kinase inhibitor that has an anticancer role by effectively inhibiting vascular endothelial growth factor receptor (VEGFR), platelet-derived growth factor receptor (PDGFR), fibroblast growth factor receptor (FGFR), and tyrosine kinase (c-Kit) (Qin et al., 2020; Lu et al., 2021; Ye et al., 2021). The ALTER-0303 phase III randomized clinical trial confirmed the effectiveness and safety of anlotinib as a third-line treatment in patients with NSCLC (Han et al., 2018). It was reported that anlotinib could improve the survival of patients diagnosed with advanced lung cancer, either NSCLC or small-cell lung cancer (SCLC) (Li et al., 2021; Qin et al., 2021). A real-world study conducted by Zhang K. et al. (2020) also confirmed the efficacy and safety of anlotinib in advanced NSCLC patients.

Either DEB-BACE or anlotinib alone was demonstrated to be effective and relatively safe in treating advanced NSCLC patients. However, the efficacy and safety of DEB-BACE combined with anlotinib in advanced NSCLC patients have not yet been demonstrated. Thus, we hypothesized that DEB-BACE and anlotinib combination therapy might offer promising outcomes in advanced NSCLC patients. The present study aimed to investigate the efficacy and safety of DEB-BACE combined with anlotinib for the treatment of advanced NSCLC.

## Patients and Methods

### Patients

This study conformed to the guidelines of the Declaration of Helsinki and was approved by the institutional review board of our institution. Written informed consent was obtained from all patients or their families before DEB-BACE was performed. From February 2018 to August 2019, a total of 51 advanced NSCLC patients (tumor stage ≥ IIIA) who underwent DEB-BACE and anlotinib treatment were analyzed in this retrospective cohort study. The inclusion criteria were as follows: (1) histopathological diagnosis of NSCLC, (2) TNM stages III–IV, (3) age between 18 and 75 years, (4) no complications of other primary malignancies, and (5) Eastern Cooperative Oncology Group performance status (ECOG PS) ≤ 2. The exclusion criteria were as follows: (1) uncontrolled hypertension, (2) abnormal coagulation function, (3) cardiac insufficiency, (4) incomplete data, and (5) an expected survival time of less than 3 months. All patients were followed-up until death or the last follow-up until July 31, 2021.

### Therapeutic Methods

#### DEB-BACE Procedure

The Seldinger puncture technique at the right femoral artery was performed under local anesthesia. A 5-F Cobra catheter (Terumo, Japan) was then introduced through a 5-F vascular sheath, and then conventional angiography of the bronchial artery, intercostal artery, and internal thoracic artery was performed to determine the blood supply to the tumors. The microcatheter was then advanced superselectively into the tumor-feeding artery when necessary. Then, the diluent of chemotherapy drugs was slowly injected through the microcatheter. The chemotherapy regimens were pemetrexed (500–750 mg) plus cisplatin (60–90 mg) for adenocarcinoma (ADC) and gemcitabine (1,000–1,500 mg) plus cisplatin (60–90 mg) for squamous cell carcinoma (SCC). Next, CalliSpheres Beads (Jiangsu Hengrui Medicine Co., Ltd., Jiangsu, China) with diameters of 300–500 μm loaded with 60 mg pirarubicin were infused *via* the microcatheter into the feeding arteries of the tumors. The loading procedure of CalliSpheres Beads-THP was performed similarly as the procedure described by Liu et al. (2019). Finally, the feeding arteries were embolized with gelfoam particles until the stasis or near-stasis of the embolized vessel was achieved. DEB-BACE was repeated in patients without local tumor progression or serious adverse events 1 month after the first session. Patients were re-evaluated every 6–8 weeks by contrast-enhanced computed tomography (CT) after the first DEB-BACE treatment cycle. All of the patients underwent the DEB-BACE procedure approximately three to six times based on imaging examination findings and physical conditions.

#### Anlotinib Treatment Protocol

One week after DEB-BACE, all patients were given anlotinib orally with an initial dose of 12 mg/day on a 2-week-on and 1-week-off treatment schedule. Dose modifications from 12 to 8 mg were permitted when intolerable adverse events occurred. Treatment was continued until disease progression or intolerance due to adverse events.

#### Evaluation and Follow-Up

During follow-up, chest CT, brain and abdominal CT, emission computed tomography when necessary, and blood tests were performed to assess tumor response. The images were independently analyzed by two experienced radiologists. The therapeutic effect was assessed according to Response Evaluation Criteria in Solid Tumors (RECIST) version 1.1. The objective response rate (ORR) was defined as the percentage of patients who achieved a complete response (CR) or partial response (PR), and the disease control rate (DCR) was defined as the proportion of patients who achieved a CR, PR, or stable disease (SD). Overall survival (OS) was defined as the time from the beginning of the first combination treatment to death or last follow-up. The time between the first combination therapy and tumor progression or death was defined as progression-free survival (PFS). Adverse events were observed clinically during admission and assessed by telephone interview after discharge according to the Common Terminology Criteria for Adverse Events version 4.0.

#### Statistical Analysis

All data were analyzed using the statistical software SPSS 22.0. Categorical variables were expressed as numbers or percentages (%), and continuous variables were expressed as the mean ± standard deviation (SD) or median (25th–75th percentiles) as appropriate. Kaplan–Meier survival curves were used to calculate OS and PFS. Univariable and multivariable Cox proportional hazards regression analyses were used to predict prognostic factors of PFS and OS. Statistical significance was defined as *P* < 0.05.

## Results

### Patient Characteristics

A total of 51 patients with advanced NSCLC were involved in the present study. The mean age of the patients was 61.4 ± 7.5 years. Forty (78.4%) patients were men, and most (76.5%) had a smoking history. The histological subtypes were squamous cell carcinoma in 17 patients (33.3%) and adenocarcinoma in 34 patients (66.7%). Of them, 28 (54.9%) patients had endothelial growth factor receptor (EGFR) mutations. All of the patients underwent the DEB-BACE procedure approximately four to six times, and all of them received anlotinib at a dose of 12 mg per day. Only eight (15.7%) patients had a reduction in anlotinib as a result of the drug not being well-tolerated. Detailed characteristics of the patients are listed in Table 1.

**TABLE 1:** Baseline characteristics.

Parameters	Value
Age (years)	61.4 ±7.5
**Sex (n/%)**	
Male	40 (78.4)
Female	11 (21.6)
**Smoking history**	
Yes	39 (76.5)
No	12 (23.5)
**ECOG performance status (n/%)**	
0-1	33 (64.7)
2	18 (35.3)
**TNM stage (n/%)**	
III	40 (78.4)
IV	11 (21.6)
**Pathology**	
Adenocarcinoma	34 (66.7)
Squamous cell carcinoma	17 (33.3)
**EGFR status**	
Mutation	28 (54.9)
Wild type/unknown	23 (45.1)
**Liver metastases**	
Yes	10 (19.6)
No	41 (80.4)
**Brain metastases**	
Yes	8 (15.7)
No	43 (84.3)

*TNM, Tumor Node Metastasis; ECOG, Eastern Cooperative Oncology Group; EGFR, endothelial growth factor receptor.*

### Clinical Efficacy

**FIGURE 1:**
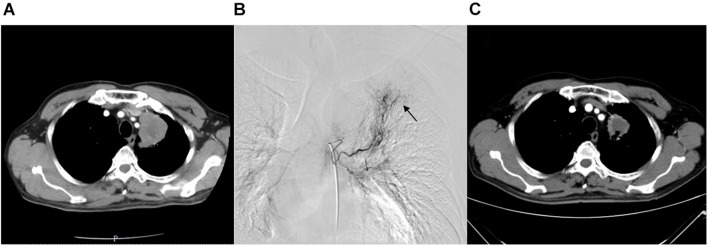
A 68-year-old female with advanced lung squamous carcinoma underwent the combination therapy of DEB-BACE and anlotinib. **(A)** Chest CT revealed a mass in the left lung superior lobe before treatment. **(B)** During the DEB-BACE procedure, the tumor was fed by the left bronchial artery (black arrow). **(C)** The patient achieved a PR after 1 month of combination therapy. DEB-BACE, drug-eluting bead bronchial arterial chemoembolization; CT, computed tomography; PR, partial response.

**FIGURE 2:**
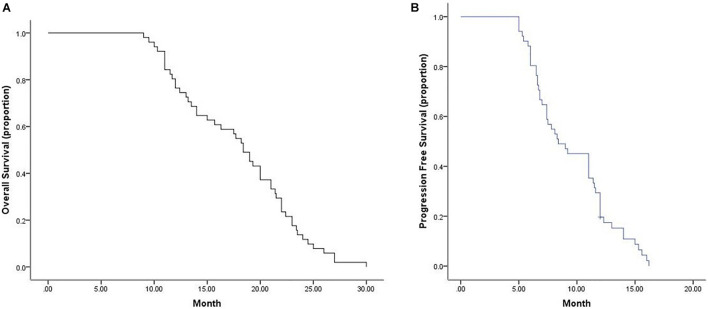
Median OS and PFS for all patients. **(A)** Graph indicates a median OS of 18.4 months (95% CI, 16.6–20.2 months). **(B)** Graph indicates a median PFS of 8.4 months (95% CI, 6.2–10.6 months). OS, overall survival; PFS, progression-free survival.

Tumor response was analyzed according to the first follow-up CT 30 days after the first combination therapy. No patients achieved a CR, 11 patients achieved a PR, 40 patients achieved SD, and no patients developed progressive disease (PD) (Figure 1); thus, the ORR was 21.6%, and the DCR was 100%. The Kaplan–Meier curves for OS and PFS are shown in Figure 2. The median OS was 18.4 months (95% CI, 16.6–20.2 months), and the median PFS was 8.4 months (95% CI, 6.2–10.6 months). Univariable Cox proportional hazard regression analysis indicated that TNM stage (IV vs. III) and brain metastases (yes vs. no) were both correlated with shorter OS and shorter PFS (*P* < 0.001, both). Additionally, EGFR status (wild type vs. mutation) was associated with shorter PFS (*P* = 0.003). Multivariable Cox regression showed that TNM stage and brain metastasis (*P* < 0.01, all) independently predicted shorter OS and PFS in patients with advanced NSCLC (Table 2).

**TABLE 2:** Factors affecting OS and PFS.

Parameters	OS				PFS			
	HR	95% CI		P	HR	95% CI		P
		Lower	Higher			Lower	Higher	
**Univariable Cox regression**								
Age (> 65 vs. ≤ 65 y	1.275	0.716	2.270	0.409	0.898	0.507	1.588	0.710
Sex (Male vs. Female)	0.565	0.281	1.136	0.109	1.009	0.500	2.037	0.981
Smoking history (Yes vs. No)	1.704	0.868	3.345	0.122	1.026	0.520	2.024	0.940
ECOG PS (2 vs. 0-1)	1.742	0.959	3.163	0.068	1.583	0.877	2.856	0.127
TNM stage (IV vs. III)	7.110	3.121	16.197	**<0.001**	5.370	2.524	11.425	**<0.001**
Pathology (Squamous vs. Adenocarcinoma)	1.094	0.602	1.987	0.769	1.075	0.590	1.958	0.814
EGFR status (Wild type vs. Mutation)	1.752	0.994	3.008	0.052	2.394	1.335	4.294	**0.003**
Liver metastases (Yes vs. No)	1.110	0.545	2.260	0.774	1.225	0.609	2.460	0.570
Brain metastases (Yes vs. No)	24.647	7.496	81.034	**<0.001**	6.492	2.652	15.894	**<0.001**
**Multivariable Cox regression**								
EGFR status (Wild type vs. Mutation)	1.20	0.958	3.086	0.069	2.498	1.368	4.560	0.003
TNM stage (IV vs. III)	4.915	1.928	12.530	**0.001**	4.105	1.728	9.752	**0.001**
Brain metastases (Yes vs. No)	25.242	6.261	101.770	**<0.001**	5.633	2.042	15.538	**0.001**

*OS, overall survival; HR, hazard ratio; CI, confidence interval; PFS, progression-free survival. The bold values are less than 0.05.*

### Adverse Events

All detailed treatment-related adverse events (TRAEs) are reported in Table 3. The most common adverse events related to DEB-BACE were nausea and vomiting, liver dysfunction, bone marrow suppression, pain, and fever. Typical complications related to oral anlotinib were fatigue, hand-foot syndrome, anorexia, and hypertension. Among the patients, there were 18 cases (35.3%) of nausea, 14 cases (27.5%) of pain, 9 cases (17.6%) of bone marrow suppression, 16 cases (31.4%) of fever, 8 cases (23.7%) of hepatic function abnormalities, 8 cases of anorexia (15.7%), 8 cases of hand-foot skin reactions (15.7%), 14 cases (27.5%) of fatigue, 15 cases (25.5%) of hand-foot syndrome, and 13 cases (23.7%) of hypertension. Grade 3 TRAEs occurred in eight patients (15.7%): three people had anorexia, three had hand-foot syndrome, and two had hypertension. A reduced dose of anlotinib was thus required in these eight patients. For all included patients, TRAEs were effectively controlled with symptomatic treatment or a reduction in the drug dosage, and no toxicity-induced deaths occurred in this study.

**TABLE 3:** Adverse reactions.

Adverse reactions n (%)	G1-2	G3	G4
Abdominal pain	14 (27.5)	−	−
Fever	16 (31.4)	−	−
Nausea	18 (35.3)		
Vomiting	7 (13.7)	−	−
Bone marrow suppression	9 (17.6)	−	−
Hepatic function abnormal	8 (15.7)	−	−
Hand-foot syndrome	15 (25.5)	3 (5.9)	−
Fatigue	14 (27.5)	−	−
Hypertension	13 (23.7)	2 (3.9)	−
Anorexia	12 (23.5)	3 (5.9)	−
Diarrhea	4 (7.8)	−	−
Proteinuria	5 (9.8)	−	−
Rash	4 (7.8)	−	−
Stomatitis	2 (3.9)	−	−
Dysphonia	3 (5.9)	−	−

## Discussion

DEB-BACE treatment showed a higher ability to prolong survival and a higher treatment response than conventional chemotherapy, which has been demonstrated by many studies (Bie et al., 2019; Lin et al., 2021; Liu et al., 2021). However, the local hypoxic environment caused by embolization is a risk factor for tumor recurrence and can arouse neovascularization in the tissue surrounding the tumors. Recently, a new type of small-molecule tyrosine kinase inhibitor involved in the VEGF, PDGF, FGFR, and c-Kit pathways, anlotinib, was approved for the treatment of NSCLC. Anlotinib was recommended as the third-line or further-line treatment for NSCLC and showed a significant effect on prolonged survival and tolerable toxicity. Many scholars recommend anlotinib as the first- or second-line treatment, not only the third- or further-line treatment regimen (Zhu et al., 2021). Zhong et al. (2020) confirmed that PFS was longer when anlotinib was used in first-/second-line treatment than in third- or further-line treatment of advanced NSCLC.

The synergistic effect of chemotherapy and targeted drugs is promising. Presently, the combination therapy of chemotherapy and anlotinib is effective and widely used in clinical practice. Zhang C. et al. (2020) reported the case of a patient with lung adenocarcinoma with brain metastasis treated with chemotherapy drugs plus anlotinib who achieved PFS of more than 2 years. Wang et al. (2020). demonstrated that NSCLC patients in the anlotinib combined with chemotherapy group achieved a better DCR (78% vs. 51%) and longer median PFS (5.0 vs. 3.5 months) than those in the chemotherapy alone group. To the best of our knowledge, evidence concerning the efficacy and safety of DEB-BACE combined with anlotinib for advanced NSCLC is still lacking. Hence, in the present study, we aimed to assess the efficacy and safety of this combination therapy in advanced NSCLC patients. A total of 51 advanced NSCLC patients were retrospectively analyzed, of whom 34 patients had adenocarcinoma and 17 had squamous cell carcinoma. The ORR and DCR were 21.6 and 100%, respectively; these rates were higher than those of the patients in the 0303 clinical trial study (9.2 and 81%, respectively) (Han et al., 2018). The DCR (100%) in the present study was much higher than that of anlotinib monotherapy (67.5%) and pemetrexed monochemotherapy (65.5%) in elderly advanced NSCLC patients (Zhu et al., 2021). In addition, the median PFS was 8.4 months, which was longer than that of DEB-BACE treatment (7.4 months) (Liu et al., 2021) and that of anlotinib monotherapy in advanced NSCLC patients (5 months) (Wu et al., 2019). A slight survival benefit could be seen in our study, with a median OS of 18.4 months, when compared with the study reported by Bie et al. (2019), who found an OS of 16.5 months using DEB-BACE monotherapy. Additionally, we found that the median OS of patients receiving DEB-BACE combined with anlotinib was much longer than that of advanced NSCLC patients receiving TAI (13.1 months) reported by Fu et al. (2016). There was an obvious survival advantage compared with anlotinib monotherapy in advanced NSCLC patients, with an OS of 10.8 months (Wang et al., 2021).

The results of this study are encouraging. The reasons are summarized as follows: on the one hand, DEB-BACE allows selective and direct intratumoral administration to mass lesions and releases the drug slowly and continuously, thereby further increasing the treatment response and reducing the occurrence of adverse reactions. In addition, DEB-BACE had extra embolization capacity, which resulted in regional cytotoxic activity and ischemia at the tumor site. On the other hand, anlotinib could effectively inhibit tumor angiogenesis under the local hypoxic environment caused by embolization (Zhong and Liu, 2021), thus having a synergistic role in the treatment of NSCLC. Combined treatment could overcome these weaknesses and improve the efficacy of monotherapy.

For DEB-BACE, the majority of adverse events were nausea and vomiting, bone marrow suppression, pain, and fever, which were mild and could be relieved by symptomatic treatment. For anlotinib, the common adverse events were fatigue, hand-foot syndrome, anorexia, and hypertension. Grade 3 TRAEs occurred in eight (15.7%) patients, in whom the dose of anlotinib had to be reduced. Overall, the adverse reactions among the patients were tolerable, and no toxicity-induced deaths occurred.

In short, the combination therapy of DEB-BACE and anlotinib demonstrated an obvious effect on the clinical treatment of advanced NSCLC patients; it could effectively prolong the OS and PFS of patients while also exhibiting well-tolerated toxicity, which may offer a promising new approach for the management of advanced NSCLC. Due to the nature of retrospective observational studies and the small sample size, future large-scale multicener prospective studies are required to verify the efficacy and safety of DEB-BACE and anlotinib in treating advanced NSCLC.

## Data Availability Statement

The original contributions presented in the study are included in the article/supplementary material, further inquiries can be directed to the corresponding author/s.

## Ethics Statement

The studies involving human participants were reviewed and approved by the institutional review board of Zhengzhou University First Affiliated Hospital. Written informed consent for participation was not required for this study in accordance with the national legislation and the institutional requirements.

## Author Contributions

JL and WZ conducted the experiments. ZL and HL collected the data. ZS and JR performed the data analysis. JL and XH wrote the manuscript. All authors contributed to the article and approved the submitted version.

## Conflict of Interest

The authors declare that the research was conducted in the absence of any commercial or financial relationships that could be construed as a potential conflict of interest.

## Publisher’s Note

All claims expressed in this article are solely those of the authors and do not necessarily represent those of their affiliated organizations, or those of the publisher, the editors and the reviewers. Any product that may be evaluated in this article, or claim that may be made by its manufacturer, is not guaranteed or endorsed by the publisher.
